# Beyond food safety: environmental and sensory drivers of organic food purchase intention in an emerging market

**DOI:** 10.3389/fnut.2026.1924574

**Published:** 2026-10-23

**Authors:** Iván Veas-González, Manuel Escobar-Farfán, Jonathan Cuevas-Lizama, Jorge Bernal-Peralta, Elizabeth Emperatriz García-Salirrosas

**Affiliations:** 1 Departamento de Administración, Facultad de Economía y Administración, Universidad Católica del Norte, Antofagasta, Chile; 2 Department of Administration, Faculty of Administration and Economics, Universidad de Santiago de Chile (USACH), Santiago, Chile; 3 Department of Administration, Faculty of Economic and Administrative Sciences, Universidad Católica de la Santísima Concepción, Concepción, Chile; 4 Facultad de Administración y Economía, Universidad de Tarapacá, Arica, Chile; 5 Faculty of Management Science, Universidad Autónoma del Perú, Lima, Peru

**Keywords:** attitude, dietary behavior, environmental concern, food safety concern, nutritional content, organic food, purchase intention, sensory appeal

## Abstract

**Background/objectives:**

Food perception encompasses a multidimensional evaluation of sensory, nutritional, safety, and sustainability attributes, yet the relative contribution of each dimension to attitude formation and subsequent purchase intention remains understudied in emerging market contexts. This study examines the relationships between five perceptual dimensions (food safety, naturalness, nutritional value, environmental concern, and sensory appeal) and attitudes toward organic food and purchase intention.

**Methods:**

Data were collected via an online survey from 255 consumers using a non-probabilistic convenience and snowball sampling approach. Partial least squares structural equation modeling with 5,000 bootstrap subsamples was employed to test six hypothesized relationships.

**Results:**

Five of the six hypothesized relationships were supported. Environmental concern, natural content, sensory appeal, and nutritional content were significantly associated with attitude, while food safety concern showed no significant association. The five perception dimensions jointly explained 64.5% of the variance in attitude. Attitude was strongly associated with purchase intention, supporting its central role within the proposed model.

**Conclusion:**

Food perception operates through an integrated, multidimensional assessment rather than single attributes. The findings highlight the relevance of considering multiple food perception dimensions when examining attitudes toward organic food and purchase-related responses.

## 1 Introduction

The global organic food market has experienced sustained expansion, with its valuation reaching approximately USD 224.71 billion in 2025, and it is projected to maintain strong growth in the coming years (1). However, this global expansion remains markedly uneven across regions. Europe represents one of the most mature organic markets, with retail sales reaching €58.7 billion in 2024 (€49.5 billion within the European Union), while several European countries have achieved organic shares exceeding 10% of total food sales. Asia has also become increasingly relevant to the global organic market, with China alone accounting for €15.5 billion in retail sales in 2024. In contrast, Africa’s organic sector remains strongly connected to international markets; more than 2.8 million hectares were managed organically in 2024, although this represented only 0.2% of the continent’s total agricultural land. Latin America and the Caribbean similarly present a distinctive market structure, combining a substantial role in organic agricultural production and international trade with a comparatively less developed documented domestic retail market. In 2024, the region exported more than 3.2 million metric tons of organic products to the European Union and the United States, accounting for 54.8% of combined organic imports into these markets (2). Taken together, these regional differences indicate that global market growth should not be interpreted as evidence of homogeneous consumer adoption. Rather, they reveal substantial disparities in the development and maturity of organic food markets across regions. In parallel, recent bibliometric evidence indicates that contemporary organic food research is increasingly centered on sustainability, consumer attitudes, purchase intentions, health consciousness, and trust (2).

Consumer attitudes have been established as one of the most robust predictors of organic food purchase intention and behavior across diverse cultural and economic contexts (3–5). Meta-analytic evidence synthesizing multiple independent studies confirms that attitude consistently exerts the largest effect on organic food purchase intentions, substantially exceeding the predictive power of subjective norms and perceived behavioral control (6). However, the upstream question of which psychological and sensory dimensions activate favorable attitude formation toward organic food has received comparatively limited attention, particularly in emerging market populations where economic constraints and cultural factors create distinct consumer dynamics (7, 8). Recent empirical studies in emerging and developing economies continue to confirm that attitude remains a central mechanism linking consumers’ health-, environmental-, and value-based evaluations to their intentions to purchase organic food. Nevertheless, positive attitudes do not always translate into purchase intention, suggesting the need to better understand the perceptual antecedents that shape favorable evaluations of organic food products (9, 10).

Recent research has moved beyond consciousness-based approaches (e.g., environmental and health consciousness) to examine how specific food perception attributes relate to attitudes and choice behavior. Previous research in Chile has primarily focused on consciousness-based antecedents of attitude, including environmental, health, and social consciousness (11), whereas the role of specific food perception attributes remains comparatively underexplored. Food perception encompasses consumers’ multidimensional evaluation of sensory, nutritional, safety, and sustainability dimensions, each of which may activate distinct psychological pathways to attitude formation (12, 13). This perspective reflects an important evolution in the organic food literature. While earlier studies primarily emphasized broad psychological orientations such as health consciousness and environmental awareness, more recent research increasingly focuses on specific food-related perceptions because they represent consumers’ direct evaluations of product attributes. These perceptions are considered more proximal determinants of attitude formation, capturing how consumers interpret tangible characteristics of organic foods before developing behavioral intentions and purchase decisions (9, 10, 14). Five dimensions emerge as particularly salient in the organic food literature: (1) food safety concern, consumers’ concern regarding chemical residues and artificial additives (15); (2) natural content, perception of absence of artificial modifications (16); (3) nutritional content, beliefs regarding nutrient density and health-promoting properties (17); (4) environmental concern, perception of ecologically sustainable production methods (18, 19); and (5) sensory appeal, perception of taste, freshness, and hedonic quality (20, 21). While each dimension has been studied in isolation, the relative contribution of these dimensions to attitude formation and the subsequent relationship between attitude and purchase intention remain understudied, particularly in emerging market contexts. Furthermore, recent reviews suggest that although food safety, health benefits, environmental sustainability, trust, and sensory quality are consistently identified as major factors associated with organic food consumption, these attributes are often examined independently rather than in tandem within an integrated perceptual framework. Consequently, the relative explanatory power of different food-perception dimensions remains poorly understood, particularly in developing economies, where consumers may evaluate organic foods through distinct psychological and contextual lenses (2).

The distinction between purchase intention and actual purchase behavior is theoretically significant in this context. Purchase intention captures consumers’ reported likelihood of future purchase at a single time point, reflecting the intentional dimension of choice without incorporating situational constraints (price, availability, product location, and competing alternatives) that attenuate the attitude-behavior relationship in real-world contexts (6, 22). This approach is particularly appropriate when the objective is to examine the psychological mechanisms underlying consumer decision-making, since it allows researchers to assess the relationships between perceptual and attitudinal factors and purchase intention while recognizing contextual variables such as product availability, certification trust, accessibility, and economic constraints that frequently characterize organic food markets in developing economies (9, 23).

The Theory of Planned Behavior (TPB) provides the theoretical foundation for examining attitude-behavior relationships. The TPB posits that behavioral intentions are determined by attitudes toward the behavior, subjective norms, and perceived behavioral control (24, 25). Attitude, defined as the degree to which an individual evaluates a behavior favorably or unfavorably, has consistently demonstrated the strongest predictive power in organic food contexts (5, 6). Recent empirical evidence continues to support the central role of attitude as the primary psychological mechanism through which consumers translate evaluations of health, environmental, sensory, and ethical product attributes into purchase-related responses. Studies conducted across diverse cultural settings have consistently shown that consumers first evaluate specific characteristics of organic foods, subsequently develop attitudinal judgments, and only then form behavioral intentions, reinforcing the theoretical relevance of positioning food perception dimensions as antecedents of attitude rather than as direct predictors of purchasing outcomes (9, 10, 14). Accordingly, the TPB framework can be extended to specify broader psychological orientations, such as food perception dimensions, as upstream determinants of attitude rather than as independent predictors of intention, an application consistent with value-belief-norm theories and extended TPB applications in sustainability consumption research (26, 27). Within this framing, the present study is positioned as an extended application of the TPB focused specifically on the attitudinal pathway, in which multidimensional food perceptions are modeled as antecedents of attitude toward organic food.

Despite substantial progress in organic food research, previous studies have predominantly focused on isolated determinants such as health consciousness, environmental concern, trust, or sensory attributes. Consequently, limited empirical evidence exists on the simultaneous assessment of multiple food-perception dimensions and their relative contributions to attitude formation. Furthermore, research examining these relationships in Latin American emerging markets remains comparatively scarce, restricting the generalizability of existing findings across different cultural and economic contexts (2, 9, 10). Importantly, the Chilean sample examined in this study is not intended to statistically represent Latin American consumers as a whole. Rather, Chile is examined as a specific national setting within the broader Latin American emerging-market context, providing context-specific evidence that contributes to the still limited understanding of organic food consumption in the region. This contextual distinction is particularly relevant because organic food consumption in emerging markets may occur under different market conditions than in more mature organic markets. Previous research indicates that organic food adoption in emerging economies may be shaped by lower consumer awareness, concerns regarding product authenticity, information asymmetries, greater price sensitivity, limited product accessibility, and differences in market maturity, all of which may influence how consumers evaluate and respond to organic food attributes (28, 29). Accordingly, this study addresses the research gap by testing an integrated structural model in which five food perception dimensions serve as antecedents of attitude toward organic food, which is associated with purchase intention. The specific research question is: To what extent are multidimensional food perception attributes (safety, naturalness, nutritional quality, environmental sustainability, and sensory appeal) associated with attitude toward organic food, and how is attitude toward organic food associated with purchase intention? By examining these relationships empirically, we aim to clarify the relative importance of different perceptual dimensions and the relationship between attitude and purchase intention.

This research contributes to the literature in three ways. First, it provides an integrative empirical examination of the relationships between five perception dimensions and attitude in a market where single-attribute research remains prevalent. Second, it specifies the relative weight of each perception dimension, providing actionable insights for marketing communication and public health messaging. Third, it extends the attitude-intention literature to an underrepresented market context, thereby advancing cross-cultural evidence on factors associated with organic food choice. Furthermore, by examining attitude as an intermediate construct linking food perceptions and purchase intention, this study provides additional theoretical support for extended TPB applications focused on the attitudinal pathway in sustainable consumption research. The findings are directly relevant to the Special Issue on ‘The Relationship Between Dietary Behavior, Food Perception and Psychological State’, as they examine the relationships among perceptual attributes, attitude as a psychological construct, and purchase intention within the context of organic food consumption.

## 2 Theoretical background

Food perception constitutes a multidimensional psychological process encompassing sensory, cognitive, and evaluative dimensions that are associated with consumer attitudes and subsequent behavioral intentions (13). While the Theory of Planned Behavior (TPB) establishes attitude as a central construct linking beliefs and behavioral intentions, the upstream dimensions that are associated with attitude formation (particularly in food consumption contexts) have received comparatively limited scholarly attention in emerging market settings. Recent bibliometric evidence indicates that consumer attitudes, health consciousness, trust, purchase intentions, and sustainability remain among the dominant themes in contemporary organic food research, while important knowledge gaps persist regarding the combined relationships of multiple food perception dimensions with consumer decision-making (2). The present study addresses this gap by examining five distinct perception attributes in relation to attitude, and the relationship between attitude and purchase intention.

### 2.1 Food safety concern and attitude toward organic food

Concerns regarding food safety encompass consumers’ interests in product composition, genetic modification status, and the presence of artificial additives, including colorants, pesticides, and synthetic preservatives (30). Food safety concerns reflect both utilitarian health considerations and identity signaling; consumers who prioritize safety seek to construct self-images as discerning purchasers committed to health protection and family welfare (15). This concern serves as a cognitive precursor to forming a favorable attitude, providing a rational justification for a preference for organic food grounded in risk-mitigation logic.

Consumers consistently perceive organic food as natural, chemical-free, and inherently safer than conventionally produced alternatives, thereby reducing health risks associated with exposure to synthetic chemicals (7, 16). Recent studies indicate that health and wellness considerations continue to shape favorable evaluations of organic food products (31). Research across diverse cultural contexts confirms that food safety concerns significantly predict favorable attitudes toward organic products (15, 22, 32, 33). Particularly in emerging markets, where health awareness intersects with trust in alternative food systems, perceptions of safety generate strong attitudinal responses. The mechanism is notably robust in family-oriented contexts where consumers extend safety concerns to dependents.

*H1*: Food safety concern perception is positively associated with attitude toward organic food.

### 2.2 Natural content and attitude toward organic food

Natural content refers to the documented absence of artificial chemical intervention, including synthetic fertilizers, insecticides, pesticides, and genetically modified organisms (GMOs) (8, 34). Consumers invoke naturalness as a quality signal that contrasts sharply with conventional production systems, which are perceived as chemically intensive. This perception operates through both epistemic (knowledge-based) and affective pathways: consumers believe that natural food is inherently superior and derive emotional satisfaction from consumption that is congruent with sustainability values (16).

Naturalness is simultaneously linked to social consciousness dimensions insofar as consumers perceive natural food as supporting community welfare and the viability of local producers (35). Trust in local producers is positively associated with perceptions of food as fresh and unmodified, suggesting a link between naturalness perception and attitude when social motivation is present. Recent research indicates that perceptions of natural content function as an important quality cue that reinforces consumer trust and strengthens favorable evaluations of organic food products (36). Empirical evidence indicates that naturalness is a significant determinant of organic food purchase decisions across German, Asian, and Latin American markets (36), suggesting cross-cultural robustness.

*H2*: Natural content perception is positively associated with attitude toward organic food.

### 2.3 Nutritional content and attitude toward organic food

Nutritional content encompasses the quantity and bioavailability of micronutrients and phytonutrients present in food products, including vitamins, minerals, antioxidants, and other health-promoting compounds (37). Organic products are commonly marketed and evaluated by consumers as nutritionally superior to conventional alternatives, a distinction marketing claims attribute to regenerative soil practices and reduced chemical residue. Empirical nutrient analyses document higher antioxidant concentrations in some organically produced items, though evidence varies by crop and region (37).

Critically, consumers appear to rely on perceived rather than objective nutritional superiority, particularly in contexts where nutritional information is limited (18). This perception-based mechanism is associated with positive attitudes through two routes: a direct belief that organic is more nutritious, and an inferred superiority stemming from the naturalness and care in production associated with organic methods. Health-conscious consumers integrate nutritional benefit beliefs into their purchase evaluations, with these beliefs having a stronger effect in high-health-involvement populations (8, 38). Recent findings indicate that health consciousness remains one of the most influential drivers of favorable evaluations and purchase intentions toward organic food products, reinforcing the importance of perceived nutritional value in consumer decision-making processes (14).

*H3*: Nutritional content perception is positively associated with attitude toward organic food.

### 2.4 Environmental concern and attitude toward organic food

Environmental concern refers to consumers’ perceived concern regarding the ecological consequences of agricultural production methods, manifesting as awareness of biodiversity loss, soil degradation, water contamination, and chemical accumulation in ecosystems (27, 39). Organic agriculture explicitly avoids synthetic pesticides and fertilizers, implements crop rotation and cover cropping to maintain soil health, and employs integrated pest management, creating a production framework perceived by consumers as ecologically benign (40). This alignment provides cognitive justification for a favorable attitude: environmentally concerned consumers perceive organic food as materially aligned with their values.

Environmental concern additionally provides affective gratification independent of instrumental benefits. Consumers report emotional satisfaction described as guilt reduction, moral alignment, or positive identity expression from purchasing food produced through ecological stewardship (8, 13). This affective-cognitive synthesis strengthens attitude formation beyond utilitarian calculation. Recent evidence further confirms that environmental awareness strengthens favorable attitudes toward organic food and promotes consumers’ willingness to engage in sustainable food consumption practices (14). Empirical evidence confirms environmental concern as a robust cross-cultural predictor of attitudes toward organic food, with the magnitude of the effect intensifying as ecological issues gain salience in public discourse (18, 19, 41).

*H4*: Environmental concern is positively associated with attitude toward organic food.

### 2.5 Sensory appeal and attitude toward organic food

Sensory attributes encompass the taste, aroma, texture, color, and overall palatability of food products as directly experienced or anticipated by consumers (20, 21). These hedonic dimensions operate as primary drivers of food choice, functioning alongside utilitarian considerations such as health and sustainability (42). Organic foods benefit from consumer beliefs that careful production methods and the absence of artificial additives result in superior sensory qualities, particularly for produce items valued for freshness perception (43).

Consumer perception that organic items taste better, smell fresher, and have superior texture is distinct from objective organoleptic measurement and appears to reflect a combination of expectancy effects and genuinely differentiated production practices (13). Perception operates through cognitive elaboration (the belief that production care improves quality) and direct sensory evaluation (experienced taste and texture superiority). Notably, sensory appeal exerts a particular influence on attitudes among younger, less health-focused consumers, suggesting it serves as a gateway attribute that activates purchase consideration independent of health or environmental motivation (35).

*H5*: Sensory appeal perception is positively associated with attitude toward organic food.

### 2.6 Attitude and purchase intention

Within the Theory of Planned Behavior framework, attitude toward a behavior represents the individual’s overall evaluation of that behavior as favorable or unfavorable (25), and constitutes the dominant antecedent of behavioral intention across consumer contexts (24, 44). Specifically for organic food consumption, meta-analytic synthesis confirms that attitude consistently exerts the largest effect on purchase intention relative to subjective norms and perceived behavioral control, with cross-cultural validation across Western, Asian, and emerging-market populations (5, 6).

Recent empirical studies continue to support the central role of attitude in explaining consumers’ purchase intentions toward organic food, suggesting that favorable evaluations of organic products remain a key determinant of behavioral responses across different cultural and market contexts (45). Nevertheless, recent studies suggest that favorable attitudes do not always translate into actual purchasing behavior, highlighting the persistence of an attitude–behavior gap in organic food consumption. This finding has reinforced scholarly interest in identifying the perceptual, cognitive, and contextual factors that contribute to attitude formation and subsequently influence purchase-related responses (9).

Purchase intention refers to consumers’ self-reported willingness, intention, and consideration to purchase organic food products. Accordingly, the construct captures a prospective behavioral disposition rather than actual or observed purchasing behavior. This distinction is important because purchase intentions may not necessarily translate into actual purchases due to contextual constraints such as price, product availability, accessibility, and competing alternatives. Nevertheless, purchase intention remains a theoretically relevant proximal outcome of attitude within consumer decision-making models and provides insight into consumers’ predisposition toward future organic food purchases (7, 23). The attitude-intention relationship remains robust, although purchase intention does not necessarily translate into actual purchasing behavior because of contextual and behavioral constraints. This measurement approach is widely implemented in early-stage market research and cross-national consumer behavior studies (3, 6).

*H6*: Attitude is positively associated with purchase intention toward organic food.

### 2.7 Proposed model

The integrated model proposes five dimensions of food perception (food safety concern, natural content, nutritional content, environmental concern, and sensory appeal) as simultaneous antecedents of attitude. Attitude is positioned as the endogenous construct linking perception-based evaluations to purchase intention. This path structure emphasizes attitude’s integrative role in the relationship between multidimensional product perceptions and purchase intention. The model is primarily grounded in an extended application of the Theory of Planned Behavior (25) focused on the attitudinal pathway, while attribute-based consumer decision-making frameworks (13) provide complementary theoretical support for examining the relationships among perception attributes, attitude, and purchase intention (Figure 1).

**Figure 1:**
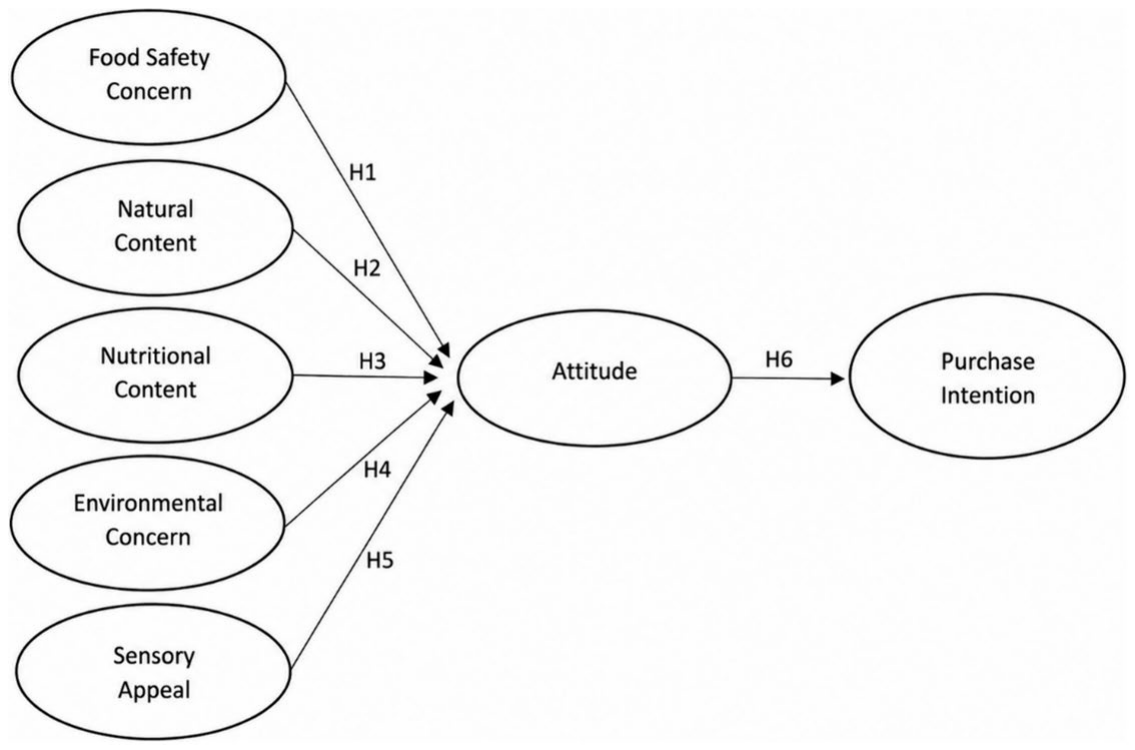
Proposed theoretical model.

## 3 Materials and methods

### 3.1 Sample

This study adopts a quantitative cross-sectional design grounded in the hypothetico-deductive approach to examine the hypothesized relationships among food perception dimensions, attitude, and organic food purchase intention. Given its explanatory and predictive nature, Partial Least Squares Structural Equation Modeling (PLS-SEM) was employed, a technique well-suited for examining relationships among latent constructs, testing hypotheses, and maximizing the explained variance of dependent variables (46, 47). This approach is consistent with prior research that has examined decision-making processes in sustainable consumption contexts (48, 49).

Data were collected during November 2024 through a survey administered via Google Forms. The target population comprised individuals aged 18 years or older residing in Chile who actively participate in food purchasing. The study was conducted in accordance with prevailing ethical principles and received approval from the Ethics Committee of Universidad Católica del Norte (protocol code 086, October 4, 2024). All participants provided informed consent prior to completing the questionnaire.

A non-probabilistic sampling strategy was employed, combining convenience sampling and snowball sampling techniques. Convenience sampling enabled the selection of participants based on their accessibility and willingness to participate, while the snowball technique facilitated sample expansion through participants’ own social networks, thereby increasing both sample size and response rate (50, 51). A total of 264 responses were collected, of which 255 were deemed valid. The remaining nine responses were excluded because participants had not provided informed consent or did not meet the eligibility criteria, particularly the minimum age requirement of 18 years. The final sample size was verified through a statistical power analysis conducted using G*Power 3.1.9.7, specifying a multiple linear regression with an effect size of f^2^ = 0.10, α = 0.05, and six predictors, yielding an observed statistical power of 0.979 (52), which substantially exceeds the conventional threshold of 0.80. Furthermore, the PLS-SEM ten-times rule was satisfied, which recommends a minimum of ten observations per incoming path to the construct with the greatest number of predictors (46).

Regarding the sociodemographic characteristics of the sample, a slight male predominance was observed (53%) relative to female respondents (45%). In terms of age distribution, there was a marked concentration within the 18–25 age bracket (59%), with limited representation of older age groups. With respect to income level, a low socioeconomic profile prevailed, with 64% of respondents reporting monthly earnings below CLP $500,000 and 23% falling within the CLP $500,001–$1,200,000 range. Finally, the majority of participants had no children (73%), with households comprising one or more dependents representing a minority (see Table A1).

### 3.2 Measurement instrument

The measurement instrument was developed based on scales previously validated in the literature on organic food consumption. A structured questionnaire consisting of 24 items was designed, each adapted from prior studies in the field. The items corresponding to the Food Safety Concern (FSC) construct were obtained from Michaelidou and Hassan (15), while those for Natural Content (NaC) and Sensory Appeal (SA) were drawn from Molinillo et al. (35). The Nutritional Content (NuC) items were obtained from Tandon et al. (53), the Environmental Concern (EC) items were taken from Jeyakumar Nathan et al. (54), the Attitude (ATT) items were adapted from Kumar et al. (55), and the Purchase Intention (PI) items were obtained from Talwar et al. (22). All items were measured using a seven-point Likert scale (1 = “strongly disagree”; 7 = “strongly agree”), enabling the assessment of respondents’ level of agreement with each statement.

### 3.3 Common method bias

Since the study relies on a cross-sectional design with data collected from a single self-report source, potential common method bias (CMB) was addressed through both procedural and statistical measures. On the procedural side, respondent confidentiality was ensured, and previously validated scales with clearly worded items were employed (56, 57). Statistically, the full-collinearity VIF criterion proposed by Kock (63) was used as a *post hoc* diagnostic, yielding values below the 3.3 threshold (maximum = 3.129). In addition, we conducted Harman’s single-factor test by entering all measurement items into an unrotated exploratory factor analysis. The first factor accounted for 48.54% of the total variance, below the commonly used 50% threshold, indicating that no single factor accounted for the majority of the variance. Taken together, these complementary diagnostics suggest that CMB is unlikely to represent a dominant source of variance in the present data. Nevertheless, given the single-source, cross-sectional, self-report design, the possibility of common method bias cannot be completely excluded.

### 3.4 Analytical approach

Data analysis and hypothesis testing were conducted using structural equation modeling (SEM), specifically through the partial least squares approach (PLS-SEM), which is recommended in the literature for predictive analysis of complex models with latent variables (56, 59). The analysis followed a two-stage procedure as recommended in the PLS-SEM literature (46). First, the measurement model was assessed using reliability and validity indicators, including factor loadings, composite reliability, and average variance extracted (AVE) (46, 57). Second, the structural model was examined using path coefficients and explained variance to evaluate the significance and direction of the relationships proposed in the model (46). The statistical significance of the coefficients was estimated using bootstrap resampling procedures with 5,000 subsamples. Additionally, potential multicollinearity was assessed using the variance inflation factor (VIF), and the overall model fit was evaluated using the SRMR index. All analyses were performed using SmartPLS 4.0 (58).

## 4 Results

### 4.1 Measurement model assessment

The measurement model evaluation indicates satisfactory psychometric properties across all variables (Table 1). In particular, the outer loadings of the indicators were consistently high, ranging from 0.758 to 0.961, thereby supporting indicator reliability. Internal consistency was strong across all constructs: Cronbach’s α ranged from 0.897 to 0.956, and composite reliability (CR) ranged from 0.924 to 0.972, all well above the 0.70 benchmark. Convergent validity was also confirmed. The average variance extracted for all latent variables was 0.709 to 0.919, exceeding the minimum acceptable level of 0.50 (60). Overall, these findings strongly support the measurement model’s adequacy with respect to reliability and convergent validity.

**Table 1:** Measurement model: factor loadings, reliability, and convergent validity.

Construct	Indicator	Loading	α	CR	AVE
Food Safety Concern (FSC)	FSC1	0.873	0.897	0.936	0.831
	FSC2	0.942			
	FSC3	0.918			
Natural Content (NaC)	NaC1	0.956	0.943	0.963	0.898
	NaC2	0.953			
	NaC3	0.933			
Nutritional Content (NuC)	NuC1	0.928	0.942	0.959	0.853
	NuC2	0.945			
	NuC3	0.954			
	NuC4	0.866			
Environmental Concern (EC)	EC1	0.878	0.897	0.924	0.709
	EC2	0.758			
	EC3	0.855			
	EC4	0.857			
	EC5	0.858			
Sensory Appeal (SA)	SA1	0.916	0.929	0.955	0.876
	SA2	0.955			
	SA3	0.935			
Attitude (ATT)	ATT1	0.955	0.956	0.972	0.919
	ATT2	0.961			
	ATT3	0.960			
Purchase Intention (PI)	PI1	0.939	0.917	0.948	0.858
	PI2	0.953			
	PI3	0.886			

α, Cronbach’s alpha; CR, composite reliability (ρc); AVE, average variance extracted.

Discriminant validity was examined through two complementary approaches: the Fornell–Larcker criterion and the HTMT ratio. The results presented in Table 2 show that the square root of the AVE for each construct (displayed along the diagonal) is consistently higher than the corresponding inter-construct correlation. This pattern indicates that each construct shares more variance with its own indicators than with other constructs, thus meeting the Fornell–Larcker requirement (57). Furthermore, the HTMT analysis provides additional evidence supporting discriminant validity. As shown in Table 3, the HTMT values range from 0.299 to 0.808, falling below the recommended threshold of 0.85 (60, 61). Although all HTMT values remained below the recommended threshold, comparatively higher values were observed between environmental concern and nutritional content (HTMT = 0.808) and between attitude and environmental concern (HTMT = 0.769), suggesting relatively closer empirical proximity among these constructs. Taken together, these results indicate that the constructs are adequately differentiated from one another, reinforcing the robustness of the measurement model.

**Table 2:** Discriminant validity: Fornell–Larcker criterion.

Construct	FSC	NaC	NuC	EC	SA	ATT	PI
Food Safety Concern (FSC)	**0.911**						
Natural Content (NaC)	0.541	**0.948**					
Nutritional Content (NuC)	0.518	0.545	**0.924**				
Environmental Concern (EC)	0.651	0.612	0.748	**0.842**			
Sensory Appeal (SA)	0.438	0.571	0.697	0.629	**0.936**		
Attitude (ATT)	0.533	0.650	0.688	0.715	0.677	**0.959**	
Purchase Intention (PI)	0.273	0.553	0.363	0.446	0.475	0.589	**0.926**

Diagonal values (bold) represent the square root of AVE. Off-diagonal values are inter-construct correlations.

**Table 3:** Discriminant validity: HTMT ratios.

Construct	FSC	NaC	NuC	EC	SA	ATT	PI
Food Safety Concern (FSC)							
Natural Content (NaC)	0.586						
Nutritional Content (NuC)	0.561	0.576					
Environmental Concern (EC)	0.723	0.668	0.808				
Sensory Appeal (SA)	0.480	0.610	0.745	0.688			
Attitude (ATT)	0.575	0.683	0.722	0.769	0.677		
Purchase Intention (PI)	0.299	0.595	0.391	0.493	0.515	0.628	

All values below the 0.85 threshold, confirming discriminant validity (61).

### 4.2 Structural model assessment

Once the properties of the measurement model had been verified, the structural model was evaluated. The overall fit was assessed using the SRMR index, yielding a value of 0.058, indicating an adequate fit, as it falls below the recommended threshold of 0.08 (59, 62). With regard to multicollinearity, the VIF values ranged from 1.000 to 3.129. In particular, the highest VIF corresponded to the relationship between environmental concern and attitude (VIF = 3.129), which remains below the conservative threshold of 3.3 suggested in the literature (63), although its proximity to this cutoff warrants cautious interpretation. Overall, the VIF results do not indicate problematic multicollinearity among the predictors.

The results of the structural model are presented in Table 4, whilst Figure 2 graphically illustrates the estimated relationships, including the standardized coefficients and R^2^ values for the endogenous constructs. In terms of explanatory power, the model adequately accounts for the variance in the endogenous constructs. Specifically, attitude achieves an R^2^ of 0.645, indicating a substantial level of explained variance, whilst reported purchase intention has an R^2^ of 0.347, reflecting a moderate level of explained variance, in accordance with criteria commonly accepted in the PLS-SEM literature (60).

**Table 4:** Structural model results: path coefficients and hypothesis testing.

Hypothesis	Relationship	*β*	SD	*t*-value	*p*-value	Decision
H1	FSC → ATT	0.037	0.051	0.734	0.463	Not Supported
H2	NaC → ATT	0.236	0.058	4.092	<0.001	Supported
H3	NuC → ATT	0.177	0.071	2.506	0.012	Supported
H4	EC → ATT	0.265	0.079	3.344	0.001	Supported
H5	SA → ATT	0.236	0.059	3.991	<0.001	Supported
H6	ATT → PI	0.589	0.049	12.029	<0.001	Supported

FSC, Food Safety Concern; NaC, Natural Content; NuC, Nutritional Content; EC, Environmental Concern; SA, Sensory Appeal; ATT, Attitude; PI, Purchase Intention; *β*, standardized path coefficient; SD, standard deviation. Bootstrapping with 5,000 subsamples.

**Figure 2:**
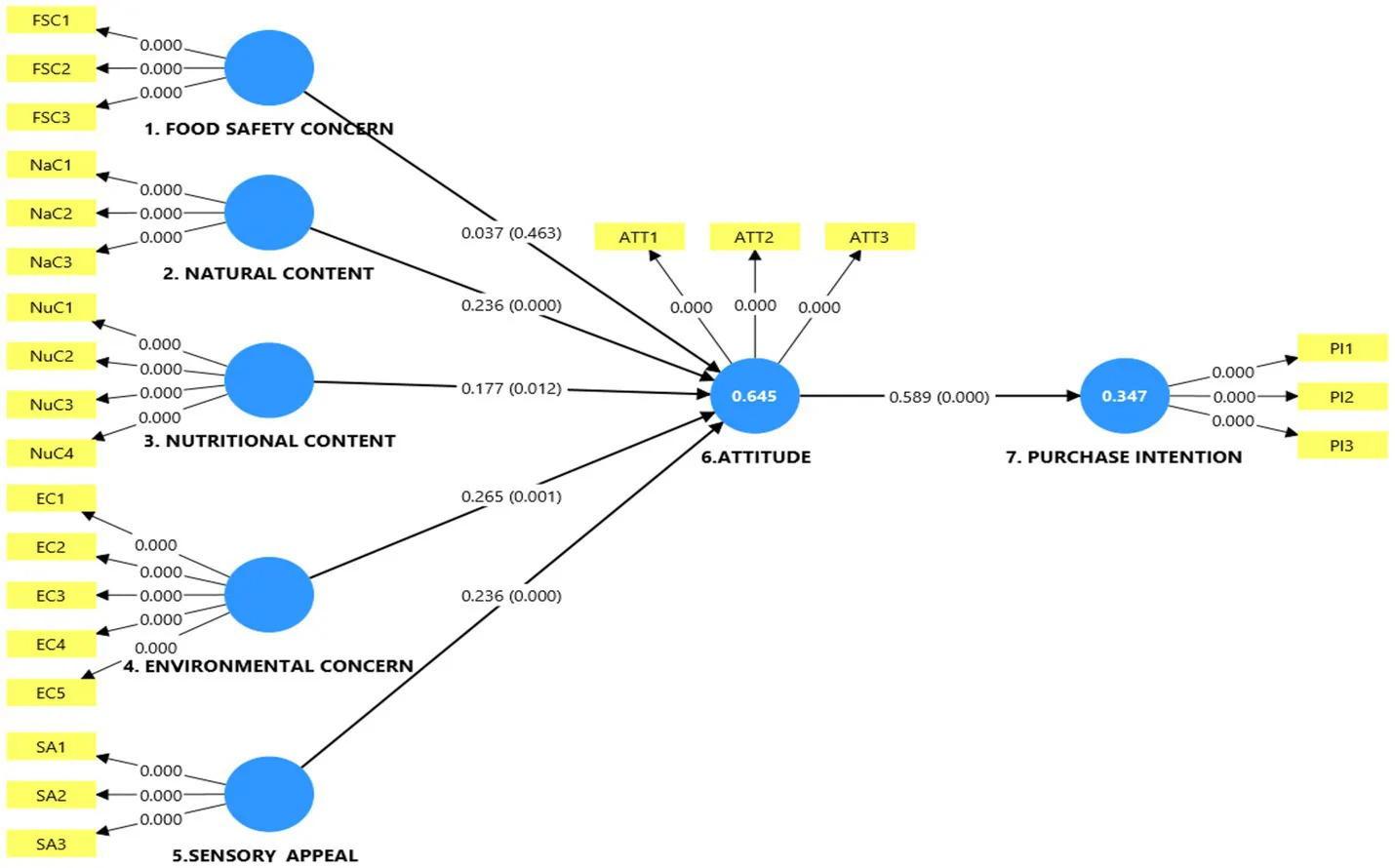
Structural model. Standardized path coefficients are shown with *p*-values in parentheses. Values in bold circles represent R^2^ for endogenous constructs.

Regarding hypothesis testing, the findings indicate that natural content (H2: *β* = 0.236, *t* = 4.092, *p* < 0.001), nutritional content (H3: *β* = 0.177, *t* = 2.506, *p* = 0.012), environmental concern (H4: *β* = 0.265, *t* = 3.344, *p* = 0.001) and sensory appeal (H5: *β* = 0.236, *t* = 3.991, *p* < 0.001) are positively and significantly associated with attitude. In contrast, food safety concern is not significantly associated with attitude (H1: *β* = 0.037, *t* = 0.734, *p* = 0.463); therefore, this hypothesis is not empirically supported. Furthermore, attitude showed a strong, positive, and statistically significant association with purchase intention (H6: *β* = 0.589, *t* = 12.029, *p* < 0.001), supporting its central role within the proposed model.

### 4.3 Predictive validity (PLS-predict)

To assess the model’s predictive power on the out-of-sample data, an analysis was conducted using the PLS-Predict procedure in SmartPLS 4 (58). As shown in Table 5, all Q^2^predict values for the indicators of the endogenous constructs were positive, indicating predictive relevance for both attitude and purchase intention. In particular, the indicators associated with attitude show higher Q^2^predict values (between 0.559 and 0.598), whilst those corresponding to purchase intention show more moderate values (between 0.232 and 0.246), although consistently above zero.

**Table 5:** PLS-predict results and predictive relevance assessment.

Endogenous construct	Indicator	Q^2^ Predict	Decision
Attitude	ATT1	0.562	Yes
	ATT2	0.598	Yes
	ATT3	0.559	Yes
Purchase Intention	PI1	0.232	Yes
	PI2	0.246	Yes
	PI3	0.243	Yes

Q^2^ predict values greater than zero indicate out-of-sample predictive relevance.

Additionally, a comparison was made between the PLS-SEM model and a linear reference model (LM). The results indicate that, whilst the linear model presents slightly lower prediction errors in some cases, the differences are not substantial, suggesting comparable performance between the two approaches. In contrast, the baseline model exhibits considerably higher errors across all indicators, reinforcing the predictive capacity of the proposed model. Taken together, these results indicate that the model exhibits moderate out-of-sample predictive capacity, in line with criteria established in recent literature, demonstrating its utility for predicting the constructs analyzed.

## 5 Discussion

This study examined how five dimensions of food perception (food safety concern, natural content, nutritional content, environmental concern, and sensory appeal) are associated with attitudes toward organic food and, subsequently, with purchase intention among Chilean consumers. Beyond identifying significant individual predictors, the findings provide evidence of the relative importance of multiple food-perception dimensions when evaluated simultaneously within an integrated model of attitude formation. This simultaneous assessment is particularly relevant because previous organic food research has frequently examined health, environmental, safety, and sensory attributes separately, making it difficult to establish their relative contribution when consumers evaluate these attributes concurrently. The results therefore extend the literature by showing that attitude toward organic food reflects a multidimensional evaluative process in which environmental, natural, sensory, and nutritional perceptions contribute with different relative strengths, rather than an evaluation dominated by a single product attribute.

Four of the five hypothesized perception-to-attitude relationships were empirically supported: natural content (*β* = 0.236, *p* < 0.001), nutritional content (*β* = 0.177, *p* = 0.012), environmental concern (*β* = 0.265, *p* = 0.001), and sensory appeal (*β* = 0.236, *p* < 0.001). The five perception dimensions jointly explained 64.5% of the variance in attitude, a level of explanatory power that substantially exceeds prior single-attribute studies. This pattern is consistent with the view that consumers consider multiple perception cues when evaluating organic food rather than relying on isolated product features. Notably, food safety concern was not significantly associated with attitude (*β* = 0.037, *p* = 0.463). This finding differs from previous studies identifying food safety concern as an important predictor of attitudes toward organic food and merits careful contextual explanation.

Three plausible contextual interpretations may help explain the non-significant relationship between food safety concern and attitude, although the present study did not directly test these mechanisms. The absence of a significant relationship should not be interpreted as indicating that food safety is unimportant to consumers. Previous research has consistently identified food safety as an important consideration in organic food evaluations (15, 32). Rather, the present findings suggest that, within this sample, food safety concern did not differentiate attitudes toward organic food to the same extent as other perception dimensions. First, environmental concern showed a larger standardized path coefficient (*β* = 0.265 vs. *β* = 0.037) and was positively correlated with food safety concern (r = 0.651), suggesting a relatively close empirical relationship between the two constructs. In the Chilean sample, environmental consciousness, which encompasses concerns about chemical residues, pesticide use, and ecosystem degradation, may overlap with concerns typically associated with personal food safety. Rather than bifurcating personal health risk from chemicals and environmental risk from chemicals, this finding may suggest that, within the present sample, environmental considerations were more salient than safety-related considerations in consumers’ evaluations of organic food. This interpretation aligns with Value-Belief-Norm theory, which posits that broader value systems (ecological stewardship) tend to subordinate more narrowly self-interested concerns (personal health protection) in attitude hierarchies. Second, food safety may represent a broadly valued attribute whose importance does not necessarily translate into greater differentiation in attitudes toward organic food. In the present sample, this may help explain why food safety concern did not differentiate between more and less favorable attitudes. Third, the demographic composition of the sample may also help contextualize this result. The respondent profile is predominantly younger and lower-income, characteristics that may be associated with greater sensitivity to price and accessibility when evaluating organic food. Within the present sample, food safety may function as a category-level assumption rather than an inter-product differentiator. Consumers do not discount safety; instead, they may assume marketplace food meets basic safety thresholds. Safety messaging may therefore have less power to distinguish attitudes toward organic versus conventional products. This interpretation aligns with evidence from other emerging market contexts (7, 8) showing that price sensitivity moderates the attitude-forming effects of health and safety benefits. These explanations should be interpreted as plausible contextual interpretations rather than empirically tested mechanisms, as the present study did not formally examine mediation, subsumption, or restricted variance as explanations for the non-significant FSC–ATT relationship.

Environmental concern, by contrast, exhibited the largest standardized path coefficient among the perception dimensions (*β* = 0.265). However, given the comparatively higher HTMT values involving environmental concern and the VIF of 3.129 for the relationship between environmental concern and attitude, this coefficient should be interpreted with caution. Although discriminant validity remained acceptable, this empirical proximity may reflect some conceptual overlap that could partly contribute to the magnitude of the environmental concern path coefficient. This pattern is consistent with evidence across cultural contexts, including meta-analytic evidence (6, 20) and studies conducted in Europe (42), Asia (19), and Latin America (10). The cross-cultural consistency suggests that ecological framing, which emphasizes soil health, biodiversity, water quality, and chemical-free production systems, may be closely associated with favorable attitudes across different income levels and regional contexts. For Chilean consumers, this salience is noteworthy because, despite organic products representing only 1% of domestic food sales, respondents demonstrated articulated environmental consciousness that was positively associated with attitudes toward organic food.

Natural content and sensory appeal emerged as co-dominant perception dimensions (both *β* = 0.236, *p* < 0.001), revealing important insights into how younger, price-sensitive consumers approach food choice. Immediate product attributes, such as the perception of chemical absence and superior sensory qualities (taste, freshness, texture), were also positively associated with attitude toward organic food, with standardized path coefficients similar in magnitude to that of environmental concern. Molinillo et al. (35) documented this pattern among Spanish millennials, noting that sensory appeal serves as a gateway attribute for purchase consideration independent of health motivation. In the Chilean context, organic products marketed with emphasis on freshness, superior taste, and visual quality may be more strongly associated with purchase consideration than abstract sustainability rhetoric. This observation carries practical implications: marketing can leverage both hedonic appeals and purity signals simultaneously rather than treating them as competing messaging strategies.

Nutritional content perception, while statistically significant, showed the smallest statistically significant standardized path coefficient among the perception dimensions (*β* = 0.177, *p* = 0.012). This comparatively weaker association may partly reflect the sample’s economic characteristics. Consumers may cognitively agree that organic is more nutritious, but this belief does not translate into proportionally stronger attitudes when price premiums represent substantial proportional costs. One possible explanation involves perceived behavioral feasibility, whereby health-benefit beliefs may be associated with weaker attitudinal responses when the behavioral option appears financially out of reach. This pattern has been documented in South Asian contexts (18, 64, 65), suggesting it represents a reliable feature of emerging markets rather than a uniquely Chilean phenomenon. Previous evidence supports this interpretation, showing that health-related evaluations continue to influence consumers’ responses toward organic food products, although their impact may be moderated by contextual and economic considerations. In particular, health consciousness remains an important driver of favorable evaluations and purchase intentions when consumers perceive organic foods as contributing to personal well-being (14).

Attitude showed a strong and statistically significant association with purchase intention (*β* = 0.589, *p* < 0.001, *t* = 12.029), corroborating meta-analytic evidence that attitude is the dominant predictor of organic food purchase intention across cultural contexts (5, 6). The magnitude of this association places it among the largest reported in recent PLS-SEM studies of organic food intention in Latin American markets. This finding is also consistent with evidence from different cultural settings, which continue to identify attitude as one of the most influential psychological mechanisms underlying consumers’ willingness to purchase organic food products (10, 45). However, the model accounts for 34.7% of the variance in purchase intention, indicating a moderate level of explanatory power and suggesting that additional psychological and situational factors may also contribute to organic food choice. It reflects a well-documented feature of intention-behavior relationships in food research: attitude is strongly associated with behavioral intention, although this relationship may be conditioned by situational constraints. Price barriers, product availability at the point of purchase, competing alternatives, and habitual purchasing patterns all attenuate the intention-behavior link in real-world contexts. This observation aligns with recent research highlighting the persistence of an attitude–behavior gap in organic food consumption, whereby favorable evaluations of organic products do not necessarily translate into actual purchasing behavior because of contextual constraints, market conditions, and consumer knowledge limitations (9). Purchase Intention, as measured in this study, captures the evaluative-intentional dimension of choice without these behavioral moderators. Future research incorporating perceived behavioral control and subjective norms would likely improve explained variance in intention and provide a more complete psychological account of organic food choice.

### 5.1 Theoretical contributions

This study makes three main theoretical contributions to the organic food and sustainable consumption literature. First, it advances a multidimensional perspective on attitude formation by simultaneously examining five food-perception dimensions (food safety concern, natural content, nutritional content, environmental concern, and sensory appeal) within a single structural model. Whereas previous studies have often examined these attributes separately, assessing them simultaneously allows researchers to evaluate their relative contributions to attitude within the same explanatory framework. The findings therefore suggest that attitudes toward organic food emerge from integrating multiple perceptual evaluations rather than from a single dominant attribute, extending attribute-based approaches to organic food decision-making.

Second, the study refines the application of the Theory of Planned Behavior by positioning multidimensional food perceptions as upstream antecedents of attitude. Rather than treating attitude as an isolated predictor of purchase-related responses, the model provides evidence about the perceptual evaluations associated with its formation. Importantly, the unequal strength of these relationships shows that different food perceptions are not equally relevant to attitude. Environmental concern showed the strongest association with attitude, followed by natural content and sensory appeal, while nutritional content showed a comparatively smaller association and food safety concern was not significantly associated with attitude when the five dimensions were considered simultaneously. This pattern suggests that organic food attitudes may reflect the joint operation of sustainability-oriented, product-related, and hedonic evaluations, thereby providing a more differentiated account of the perceptual foundations of attitude formation.

Third, the study contributes context-specific evidence from an underrepresented Latin American emerging-market setting. Rather than assuming that perception-attitude relationships established in mature organic markets are universally transferable, the findings show the value of examining these relationships under different market and socioeconomic conditions. The Chilean evidence indicates that the relative salience of organic food attributes may differ in less mature organic markets, extending the literature’s geographical scope while highlighting the importance of contextual boundary conditions in models of organic food decision-making. Accordingly, the study contributes not by claiming generalizability to Latin America as a whole, but by providing theoretically relevant evidence from a national context that remains comparatively underrepresented in the international organic food literature.

### 5.2 Managerial implications

The findings offer several practical implications for organic food producers, retailers, and marketers targeting consumer segments similar to those in the present Chilean sample, particularly younger and lower-income consumers. First, marketing strategies should consider the relative importance of the perceptual attributes associated with consumer attitudes rather than relying predominantly on food safety claims. Environmental concern showed the strongest association with attitude, while natural content and sensory appeal also showed relevant associations. Accordingly, communication strategies could combine environmental information, such as sustainable production practices, reduced chemical use, biodiversity protection, and responsible resource management, with more immediate product-related cues concerning naturalness, freshness, taste, and sensory quality. This combination may allow brands to communicate both the broader sustainability value of organic food and the tangible benefits consumers can experience directly.

Second, the findings have implications for product positioning and communication across different consumer touchpoints. Packaging, point-of-sale materials, retailer displays, and digital communication can make environmental and natural attributes more visible while simultaneously emphasizing sensory characteristics. Rather than presenting sustainability as an abstract benefit, firms could translate environmental attributes into clear and accessible product information supported by credible certification and labeling. At the same time, sensory cues such as freshness, taste, appearance, and product quality can help connect sustainability-oriented positioning with the immediate consumption experience. The comparatively smaller association of nutritional content and the non-significant association of food safety concern with attitude do not imply that these attributes are irrelevant. Rather, they suggest that relying on safety or nutritional claims alone may provide limited differentiation when consumers evaluate organic products across multiple attributes simultaneously.

Third, the findings are relevant for market segmentation and the development of organic food offerings targeting consumer segments with characteristics similar to those represented in this study. Given the demographic composition of the sample, which is predominantly young and lower-income, managers should be cautious about extending these findings to all consumer segments. Nevertheless, the results indicate that sustainability and sensory value can coexist as relevant elements in the evaluation of organic food even within a consumer segment facing greater economic constraints. For firms targeting similar segments, this suggests an opportunity to position organic products not exclusively as premium health products, but as products that combine environmental responsibility, naturalness, and enjoyable consumption experiences. Such positioning may help broaden the perceived value proposition of organic food beyond traditional health and safety narratives.

For policymakers and organizations promoting sustainable food consumption, the findings suggest that communication initiatives could complement health-oriented information with clearer explanations of the environmental attributes associated with organic production. Eco-labeling, certification schemes, consumer education, and public communication can make information about production practices, biodiversity, soil conservation, and resource use more understandable and visible to consumers. However, because this study examines perceptual associations rather than the effectiveness of specific interventions, these recommendations should be interpreted as potential communication directions that require further experimental or field-based validation.

## 6 Conclusion

This study provides evidence that food perception is multidimensionally associated with attitudes toward organic food. Environmental concern, natural content, and sensory appeal showed the largest standardized path coefficients among the perception dimensions. Nutritional content showed a positive and statistically significant association with attitude, while food safety concerns showed no statistically significant association. Attitude, in turn, was strongly and positively associated with purchase intention, supporting its central role within the proposed model. The five perception dimensions jointly explain 64.5% of attitude variance, indicating substantial explanatory power within the proposed model. These findings reinforce contemporary perspectives suggesting that consumers develop attitudes toward organic food through the integrated evaluation of multiple product attributes rather than through isolated health, environmental, or sensory considerations alone.

The lack of significance of food safety concerns challenges assumptions embedded in much of the organic food marketing and consumer behavior literature. It suggests that, within the present sample, food safety may function as a category-level baseline assumption rather than as a product-differentiating attitude cue. This raises important empirical questions about whether safety messaging becomes cognitively subsumed by broader environmental consciousness or simply loses salience in contexts where behavioral options are constrained by economic barriers.

In practice, these results suggest that marketing strategies targeting consumer segments similar to those in the present Chilean sample could complement health and safety communication by placing greater emphasis on environmental stewardship and sensory-quality messaging. Marketing resources should support the visibility of environmental certification and hedonic product framing, emphasizing taste, freshness, and visual appeal. For policymakers seeking to promote sustainable food consumption, the findings suggest that environmental and community-level information could complement individual health-benefit communication, although further empirical validation is needed to confirm the effectiveness of such approaches.

### 6.1 Limitations and future research

As with any empirical investigation, this study has inherent limitations that suggest directions for future research. The cross-sectional design captures associations at a single time point and does not establish causality. Accordingly, the structural relationships identified in this study should be interpreted as associations rather than causal effects.

Purchase Intention was assessed through self-reported items capturing respondents’ willingness, intention, and consideration to purchase organic food rather than actual or observed purchasing behavior. Accordingly, the construct should be interpreted as a prospective behavioral intention, not as evidence of realized purchase behavior. Although this operationalization aligns with the conceptualization of purchase intention adopted in this study, it does not establish whether these intentions translate into actual purchases. Future research could complement self-reported purchase intentions with observed transactions, longitudinal follow-up, or other behavioral measures to examine the extent to which intentions translate into actual organic food purchases.

Furthermore, although the statistical diagnostics did not indicate that common method bias was a dominant concern, all constructs were measured using self-reported data collected from the same respondents at a single point in time. Therefore, the possibility of common method bias cannot be completely ruled out, and some observed associations may be affected by shared method variance. Future research could address this limitation through longitudinal or multi-source designs, temporal separation of predictor and outcome measures, or incorporating behavioral data.

The structural model accounts for 34.7% of variance in purchase intention, suggesting that additional psychological and situational factors beyond those specified here influence organic food choice. The model focuses exclusively on the attitudinal pathway of the Theory of Planned Behavior, which represents a theoretical limitation of the present study. Future research incorporating subjective norms and perceived behavioral control could provide a more comprehensive account of organic food choice and examine the complementary role of normative and control mechanisms. Further studies could also integrate consumption-value or cognitive-affective frameworks to examine whether utilitarian, sustainability-oriented, and hedonic evaluations contribute to attitude formation through distinct psychological mechanisms.

The use of convenience and snowball sampling also limits the findings’ generalizability. In addition, the demographic composition of the sample, particularly its predominance of younger and lower-income respondents, means that the observed relationships should not be assumed to represent the broader Chilean population or consumers in other Latin American or emerging-market contexts. Future research should use probability-based or more demographically diverse samples to assess whether the observed relationships hold across different consumer segments and socioeconomic contexts.

Additionally, the relative importance of perception dimensions may vary across different consumer segments, geographic contexts, and market conditions; replication studies in diverse settings would test the cross-cultural robustness of these findings.

## Data Availability

The raw data supporting the conclusions of this article will be made available by the authors, without undue reservation.

## Appendix

**Table A1:** Demographic characteristics of the sample (*n* = 255).

Variable	Category	*n*	%
Gender	Male	136	53%
	Female	116	45%
	Other/Prefer not to say	3	1%
Age (years)	18–25	151	59%
	26–30	41	16%
	> 30	63	25%
Income range (CLP)	< 500,000	164	64%
	500,001 – 1,200,000	58	23%
	> 1,200,000	33	13%
Number of children	0	187	73%
	1–2	47	18%
	≥ 3	21	9%

## References

[1] Fortune Business Insights. (2026). Organic foods market size, share, growth & forecast, 2034. Available online at: https://www.fortunebusinessinsights.com/industry-reports/organic-foods-market-101470 (Accessed June 24, 2026).

[2] Azizan A Awal A Zain WZWM Hairoman N Endrini S Hassan F. Organic food research: key contributors, research hotspots, and emerging trends. Appl Food Res. (2025) 5:101109. doi: 10.1016/J.AFRES.2025.101109

[3] Ham M Pap A Stanic M. What drives organic food purchasing? – evidence from Croatia. Br Food J. (2018) 120:734–48. doi: 10.1108/BFJ-02-2017-0090

[4] Çabuk S Tanrikulu C Gelibolu L. Understanding organic food consumption: attitude as a mediator. Int J Consum Stud. (2014) 38:337–45. doi: 10.1111/ijcs.12094

[5] Scalco A Noventa S Sartori R Ceschi A. Predicting organic food consumption: a meta-analytic structural equation model based on the theory of planned behavior. Appetite. (2017) 112:235–48. doi: 10.1016/j.appet.2017.02.007, 28188865

[6] Leonidou LC Eteokleous PP Christofi A-M Korfiatis N. Drivers, outcomes, and moderators of consumer intention to buy organic goods: meta-analysis, implications, and future agenda. J Bus Res. (2022) 151:339–54. doi: 10.1016/j.jbusres.2022.06.027

[7] Pham TH Nguyen TN Phan TTH Nguyen NT. Evaluating the purchase behaviour of organic food by young consumers in an emerging market economy. J Strateg Mark. (2019) 27:540–56. doi: 10.1080/0965254X.2018.1447984

[8] Tandon A Dhir A Kaur P Kushwah S Salo J. Why do people buy organic food? The moderating role of environmental concerns and trust. J Retail Consum Serv. (2020) 57:102247. doi: 10.1016/j.jretconser.2020.102247

[9] Arslan Y. Unpacking organic food purchase intentions in an S-O-R framework: the multidimensional moderating roles of consumer knowledge and trust. Food Sci Nutr. (2025) 13:e70447. doi: 10.1002/fsn3.70447 PMC12177418 40538990

[10] Leyva-Hernández SN Toledo-López A Hernández-Lara AB Arango-Ramírez PM Avendaño-Rito M d C. Values, attitude, and desire in organic food purchase intention in Mexico. Front Sustain Food Syst. (2024) 8:1516788. doi: 10.3389/fsufs.2024.1516788

[11] Escobar-Farfán M Veas-González I Bernal-Peralta J Saavedra García T Santibáñez Labraña C. Environmental, health, and social consciousness as drivers of organic food choice. Nutrients. (2026) 18:1242. doi: 10.3390/NU18081242 PMC13118993 42075054

[12] Prentice C Chen J Wang X. The influence of product and personal attributes on organic food marketing. J Retail Consum Serv. (2019) 46:70–8. doi: 10.1016/j.jretconser.2017.10.020

[13] Lee H-J Yun Z-S. Consumers’ perceptions of organic food attributes and cognitive and affective attitudes as determinants of their purchase intentions toward organic food. Food Qual Prefer. (2015) 39:259–67. doi: 10.1016/j.foodqual.2014.06.002

[14] Li Y Shan B. Exploring the role of health consciousness and environmental awareness in purchase intentions for green-packaged organic foods: an extended TPB model. Front Nutr. (2025) 12:1528016. doi: 10.3389/fnut.2025.1528016 PMC11921780 40110167

[15] Michaelidou N Hassan LM. The role of health consciousness, food safety concern and ethical identity on attitudes and intentions towards organic food. Int J Consum Stud. (2008) 32:163–70. doi: 10.1111/j.1470-6431.2007.00619.x

[16] Aitken R Watkins L Williams J Kean A. The positive role of labelling on consumers’ perceived behavioural control and intention to purchase organic food. J Clean Prod. (2020) 255:120334. doi: 10.1016/j.jclepro.2020.120334

[17] Pan J Wu K-S Huang D-M Sun S-W. Role of health-consciousness on purchase intention of health and wellness food: the serial mediating effect of attitude and desire. Nutrients. (2025) 17:746. doi: 10.3390/nu17050746, 40077617 PMC11901681

[18] Akter S Ali S Fekete-Farkas M Fogarassy C Lakner Z. Why organic food? Factors influence the organic food purchase intension in an emerging country (study from northern part of Bangladesh). Resources. (2023) 12:5. doi: 10.3390/resources12010005

[19] Zhou Y Thøgersen J Ruan Y Huang G. The moderating role of human values in planned behavior: the case of Chinese consumers’ intention to buy organic food. J Consum Mark. (2013) 30:335–44. doi: 10.1108/JCM-02-2013-0482

[20] Massey M O’Cass A Otahal P. A meta-analytic study of the factors driving the purchase of organic food. Appetite. (2018) 125:418–27. doi: 10.1016/j.appet.2018.02.029, 29501680

[21] Magnusson MK Arvola A Koivisto Hursti U Åberg L Sjödén P. Attitudes towards organic foods among Swedish consumers. Br Food J. (2001) 103:209–27. doi: 10.1108/00070700110386755

[22] Talwar S Jabeen F Tandon A Sakashita M Dhir A. What drives willingness to purchase and stated buying behavior toward organic food? A stimulus–organism–behavior–consequence (SOBC) perspective. J Clean Prod. (2021) 293:125882. doi: 10.1016/j.jclepro.2021.125882

[23] Nguyen TT Dang HQ Le-Anh T. Impacts of household norms and trust on organic food purchase behavior under adapted theory of planned behavior. J Agribus Dev Emerg Econ. (2024) 14:1055–73. doi: 10.1108/JADEE-10-2022-0218

[24] Ajzen I. The theory of planned behavior: frequently asked questions. Hum Behav Emerg Technol. (2020) 2:314–24. doi: 10.1002/hbe2.195

[25] Ajzen I. The theory of planned behavior. Organ Behav Hum Decis Process. (1991) 50:179–211. doi: 10.1016/0749-5978(91)90020-T

[26] Kareklas I Carlson JR Muehling DD. “I eat organic for my benefit and yours”: egoistic and altruistic considerations for purchasing organic food and their implications for advertising strategists. J Advert. (2014) 43:18–32. doi: 10.1080/00913367.2013.799450

[27] Stern PC. New environmental theories: toward a coherent theory of environmentally significant behavior. J Soc Issues. (2000) 56:407–24. doi: 10.1111/0022-4537.00175

[28] Abdul Latip MS. Do dietary habits shape consumer responses to greenwashing and green communication? Evidence from the organic food market. Br Food J. (2026) 128:3574–91. doi: 10.1108/BFJ-10-2025-1342

[29] Synodinos C do Prado NB Marcondes de Moraes GHS. Is TPB still relevant for generation y’s organic food behavior? A comparative SEM and fsQCA study in South Africa. Sustainability. (2026) 18:1348. doi: 10.3390/SU18031348

[30] Teng C-C Lu C-H. Organic food consumption in Taiwan: motives, involvement, and purchase intention under the moderating role of uncertainty. Appetite. (2016) 105:95–105. doi: 10.1016/j.appet.2016.05.006, 27178878

[31] Liu P Wen H Mattila AS. Centering on health and wellbeing: examining consumers’ organic food purchasing decisions in restaurants when traveling. Int J Hosp Manag. (2025) 126:104082. doi: 10.1016/J.IJHM.2025.104082

[32] Hsu S-Y Chang C-C Lin TT. An analysis of purchase intentions toward organic food on health consciousness and food safety with/under structural equation modeling. Br Food J. (2016) 118:200–16. doi: 10.1108/BFJ-11-2014-0376

[33] Pino G Peluso AM Guido G. Determinants of regular and occasional consumers’ intentions to buy organic food. J Consum Aff. (2012) 46:157–69. doi: 10.1111/j.1745-6606.2012.01223.x

[34] Chan RYK Lau LBY. Explaining green purchasing behavior. J Int Consum Mark. (2001) 14:9–40. doi: 10.1300/J046v14n02_02

[35] Molinillo S Vidal-Branco M Japutra A. Understanding the drivers of organic foods purchasing of millennials: evidence from Brazil and Spain. J Retail Consum Serv. (2020) 52:101926. doi: 10.1016/j.jretconser.2019.101926

[36] Sarangi S Moharana R Gayathri JS. Exploring consumer perceptions and purchasing behaviors towards organic food: an empirical investigation. Br Food J. (2025) 127:1379–94. doi: 10.1108/BFJ-07-2024-0763

[37] Barański M Średnicka-Tober D Volakakis N Seal C Sanderson R Stewart GB . Higher antioxidant and lower cadmium concentrations and lower incidence of pesticide residues in organically grown crops: a systematic literature review and meta-analyses. Br J Nutr. (2014) 112:794–811. doi: 10.1017/S0007114514001366, 24968103 PMC4141693

[38] Steptoe A Pollard TM Wardle J. Development of a measure of the motives underlying the selection of food: the food choice questionnaire. Appetite. (1995) 25:267–84. doi: 10.1006/appe.1995.0061 8746966

[39] Wijekoon R Sabri MF. Determinants that influence green product purchase intention and behavior: a literature review and guiding framework. Sustainability. (2021) 13:6219. doi: 10.3390/su13116219

[40] Tsakiridou E Boutsouki C Zotos Y Mattas K. Attitudes and behaviour towards organic products: an exploratory study. Int J Retail Distrib Manag. (2008) 36:158–75. doi: 10.1108/09590550810853093

[41] Ferreira S Pereira O. Antecedents of consumers’ intention and behavior to purchase organic food in the Portuguese context. Sustainability. (2023) 15:9670. doi: 10.3390/su15129670

[42] Espejel J Fandos C Flavián C. The role of intrinsic and extrinsic quality attributes on consumer behaviour for traditional food products. Manag Serv Qual. (2007) 17:681–701. doi: 10.1108/09604520710835000

[43] Chekima B Oswald AI Wafa SAWSK Chekima K. Narrowing the gap: factors driving organic food consumption. J Clean Prod. (2017) 166:1438–47. doi: 10.1016/j.jclepro.2017.08.086

[44] Escobar-Farfán M García-Salirrosas EE Guerra-Velásquez M Veas-González I Gómez-Bayona L Gallardo-Canales R. Psychological determinants of healthy food purchase intention: an integrative model based on health consciousness. Nutrients. (2025) 17:1140. doi: 10.3390/nu17071140, 40218899 PMC11990704

[45] Eberle L Milan GS Borchardt M Pereira GM Graciola AP. Determinants and moderators of organic food purchase intention. Food Qual Prefer. (2022) 100:104609. doi: 10.1016/J.FOODQUAL.2022.104609

[46] Hair JF Jr Hult GTM Ringle CM Sarstedt M Danks NP Ray S. Partial Least Squares Structural Equation Modeling (PLS-SEM) Using R: A Workbook. Cham: Springer Nature (2021).

[47] Sarstedt M Ringle CM Hair JF. Partial least squares structural equation modeling. In: Homburg C Klarmann M Vomberg A, editors. Handbook of Market Research. Cham: Springer International Publishing (2022). p. 587–632.

[48] Khan Y Hameed I Akram U. What drives attitude, purchase intention and consumer buying behavior toward organic food? A self-determination theory and theory of planned behavior perspective. Br Food J. (2022) 125:2572–87. doi: 10.1108/BFJ-07-2022-0564

[49] Thi Nguyen NP Dang HD. Organic food purchase decisions from a context-based behavioral reasoning approach. Appetite. (2022) 173:105975. doi: 10.1016/j.appet.2022.105975, 35247477

[50] Etikan I Musa SA Alkassim RS. Comparison of convenience sampling and purposive sampling. Am J Theor Appl Stat. (2016) 5:1–4. doi: 10.11648/j.ajtas.20160501.11

[51] Naderifar M Goli H Ghaljaie F. Snowball sampling: a purposeful method of sampling in qualitative research. Strides Dev Med Educ. (2017) 14:1–6. doi: 10.5812/sdme.67670

[52] Cohen J. A power primer. Psychol Bull. (1992) 112:155–9. doi: 10.1037/0033-2909.112.1.155, 19565683

[53] Tandon A Jabeen F Talwar S Sakashita M Dhir A. Facilitators and inhibitors of organic food buying behavior. Food Qual Prefer. (2021) 88:104077. doi: 10.1016/j.foodqual.2020.104077

[54] Jeyakumar Nathan R Soekmawati VV Popp J Fekete-Farkas M Oláh J. Food innovation adoption and organic food consumerism—a cross national study between Malaysia and Hungary. Foods. (2021) 10:363. doi: 10.3390/foods10020363 PMC7915773 33562411

[55] Kumar R Tripathi V Yadav V Ashesh G Mehrotra R. Modelling the role of perceived marketplace influence and moral norms in organic food consumption: a moderated mediation approach. Manag Environ Qual. (2024) 35:1484–504. doi: 10.1108/MEQ-08-2023-0239

[56] Sarstedt M Liu Y. Advanced marketing analytics using partial least squares structural equation modeling (PLS-SEM). J Mark Anal. (2024) 12:1–5. doi: 10.1057/s41270-023-00279-7

[57] Fornell C Larcker DF. Evaluating structural equation models with unobservable variables and measurement error. J Mark Res. (1981) 18:39–50. doi: 10.2307/3151312

[58] Ringle CM Wende S Becker J-M. (2022) Bönningstedt: SmartPLS. (2022). Available online at: https://www.smartpls.com (Accessed April 28, 2026).

[59] Hair J Jr Page M Brunsveld N. Essentials of Business Research Methods. New York, NY: Routledge (2019).

[60] Hair JF Risher JJ Sarstedt M Ringle CM. When to use and how to report the results of PLS-SEM. Eur Bus Rev. (2019) 31:2–24. doi: 10.1108/EBR-11-2018-0203

[61] Henseler J Ringle CM Sarstedt M. A new criterion for assessing discriminant validity in variance-based structural equation modeling. J Acad Mark Sci. (2015) 43:115–35. doi: 10.1007/s11747-014-0403-8

[62] Hu L Bentler PM. Cutoff criteria for fit indexes in covariance structure analysis: conventional criteria versus new alternatives. Struct Equ Model. (1999) 6:1–55. doi: 10.1080/10705519909540118

[63] Kock N. Common method bias in PLS-SEM: a full collinearity assessment approach. Int J E Collab. (2015) 11:1–10. doi: 10.4018/ijec.2015100101

[64] Parashar S Singh S Sood G. Examining the role of health consciousness, environmental awareness and intention on purchase of organic food: a moderated model of attitude. J Clean Prod. (2023) 386:135553. doi: 10.1016/j.jclepro.2022.135553

[65] Hair JF Page M Brunsveld N. Essentials of Business Research Methods. 4th ed. New York: Routledge (2019).

